# Synthesis and biological activity of *N*-substituted-tetrahydro-γ-carbolines containing peptide residues

**DOI:** 10.3762/bjoc.10.13

**Published:** 2014-01-15

**Authors:** Nadezhda V Sokolova, Valentine G Nenajdenko, Vladimir B Sokolov, Daria V Vinogradova, Elena F Shevtsova, Ludmila G Dubova, Sergey O Bachurin

**Affiliations:** 1 Department of Chemistry, Moscow State University, Leninskie Gory 1, Moscow, 119992, Russia; 2 Institute of Physiologically Active Compounds, Russian Academy of Sciences, Severny proezd 1, Chernogolovka, 142432, Russia; 3 A. N. Nesmeyanov Institute of Organoelement Compounds, Russian Academy of Sciences, Vavilov str. 28, 119991 Moscow, Russian Federation

**Keywords:** mitochondrial membrane potential, mitochondrial permeability transition, multicomponent, peptides, tetrahydro-γ-carbolines, Ugi multicomponent reaction

## Abstract

The synthesis of novel peptide conjugates of *N*-substituted-tetrahydro-γ-carbolines has been performed using the sequence of the Ugi multicomponent reaction and Cu(I)-catalyzed click chemistry. The effect of obtained γ-carboline–peptide conjugates on the rat liver mitochondria was evaluated. It was found that all compounds in the concentration of 30 µM did onot induce depolarization of mitochondria but possessed some inhibitory effect on the mitochondria permeability transition. The original *N*-substituted-tetrahydro-γ-carbolines containing an terminal alkyne group demonstrated a high prooxidant activity, whereas their conjugates with peptide fragments slightly inhibited both autooxidation and the *t-*BHP-induced lipid peroxidation.

## Introduction

The design and synthesis of new efficient pharmaceutical drugs for the treatment and prevention of a wide range of neurodegenerative diseases, such as Alzheimer’s dementia, Parkinsonism and the amyotrophic lateral sclerosis, is of considerable interest in modern medicinal chemistry. Mitochondrial dysfunction was found to play a crucial role in the pathogenesis of these diseases [1–3]. Thus, one of the specific symptoms of such pathologies is a decrease in the ability of mitochondria to regulate the calcium homeostasis in cells and malfunction of mitochondrial permeability transition (MPT) that represents a key step in the cascades of cell death. From this point of view, mitochondria and the MPT process are very attractive targets for the search of new neuroprotective agents [4–5].

**Figure 1:**
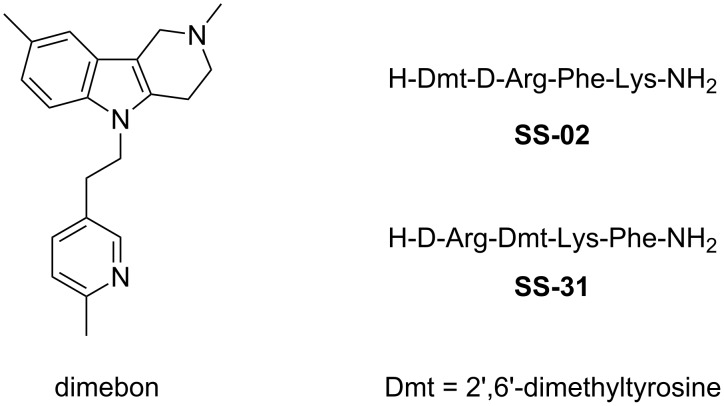
Structures of dimebon and SS peptides.

Several promising mitochondria-targeting neuroprotectors have been reported in the literature. Thus, the antihistaminic drug dimebon [6–7], which relates to tetrahydro-γ-carboline derivatives, has been found to stabilize and improve mitochondrial functions in different in vivo and in vitro models [8–9] (Figure 1). Another promising class of neuroprotectors are cell-permeable mitochondria-targeting synthetic small peptides, for example, the SS (Szeto–Schiller) peptide antioxidants [10] (Figure 1). These peptides were found to scavenge hydrogen peroxide and peroxynitrite and inhibit lipid peroxidation in vitro. By reducing mitochondrial reactive oxygen species, they inhibit MPT and cytochrome c release, thus protecting cells from oxidative cell death [11].

We expected that the conjugation of tetrahydro-γ-carbolines with synthetic peptides could lead to a new class of promising neuroprotectors affecting brain mitochondria. Thus, we synthesized N-substituted tetrahydro-γ-carbolines and their peptide conjugates and investigated the action of the obtained compounds on the mitochondria membrane potential, mitochondrial permeability transition and lipid peroxidation.

## Results and Discussion

**Scheme 1:**
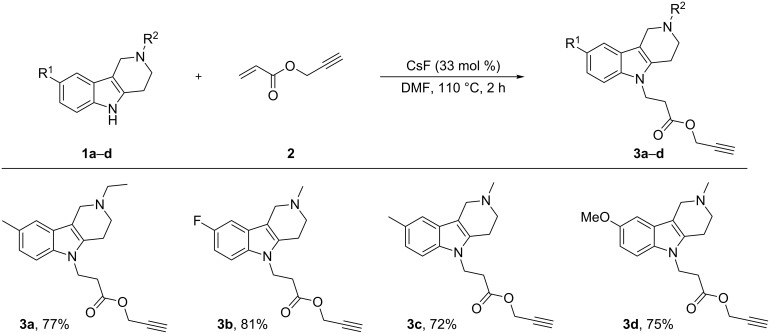
Synthesis of starting N-substituted tetrahydro-γ-carbolines **3a–d**.

The starting N-substituted tetrahydro-γ-carbolines **3a–d** containing a terminal alkyne group were prepared in good yields by heating compounds **1a–d** [12–13] with propargyl acrylate (**2**) in the presence of catalytic amounts of CsF (Scheme 1).

**Scheme 2:**
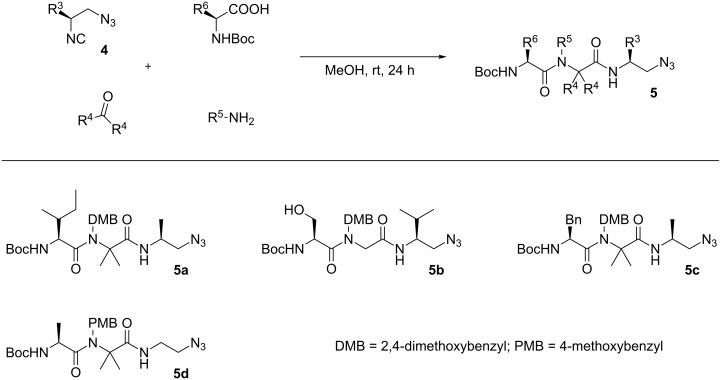
Synthesis of peptides **5** through the Ugi reaction.

The corresponding protected azidopeptides **5** [14–15] are accessible by the Ugi multicomponent reaction [16–19] of chiral isocyanoazides **4** [20] with carbonyl compounds, amines and Boc-protected amino acids (Scheme 2). As we showed before, the racemization of the chiral centre of the isocyanoazide does not occur under the conditions of the Ugi reaction [20]. This approach permits to prepare a broad variety of azidopeptides using multicomponent methodology.

**Scheme 3:**
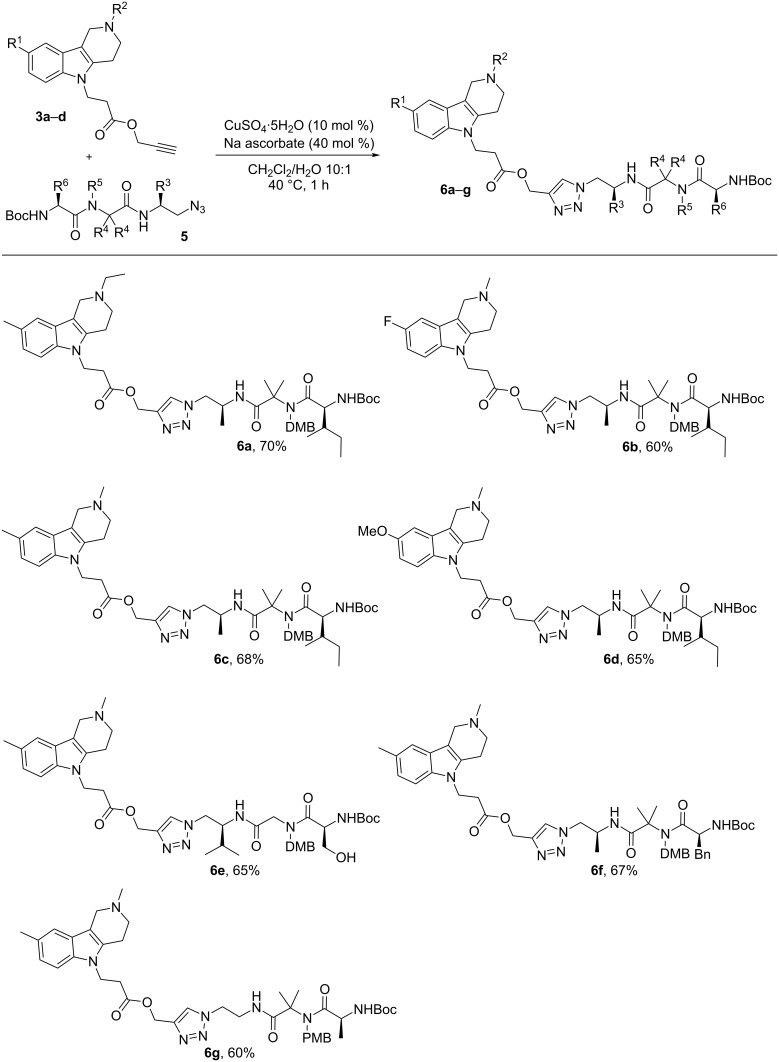
Synthesis of N-substituted tetrahydro-γ-carbolines containing protected peptide residues.

The modification of N-substituted tetrahydro-γ-carbolines **3a–d** by peptide fragments was performed using Cu(I)-catalyzed click chemistry – one of the most effective conjugation methods [21–22]. Thus, heating the educts with Cu(II)/sodium ascorbate in a biphasic mixture of CH_2_Cl_2_/H_2_O during 1 h provided compounds **6a–g** (Scheme 3). According to the ^13^C NMR spectra, the click reaction proceeds regioselective in all cases affording the desired conjugates **6a–g** in good yields.

**Scheme 4:**
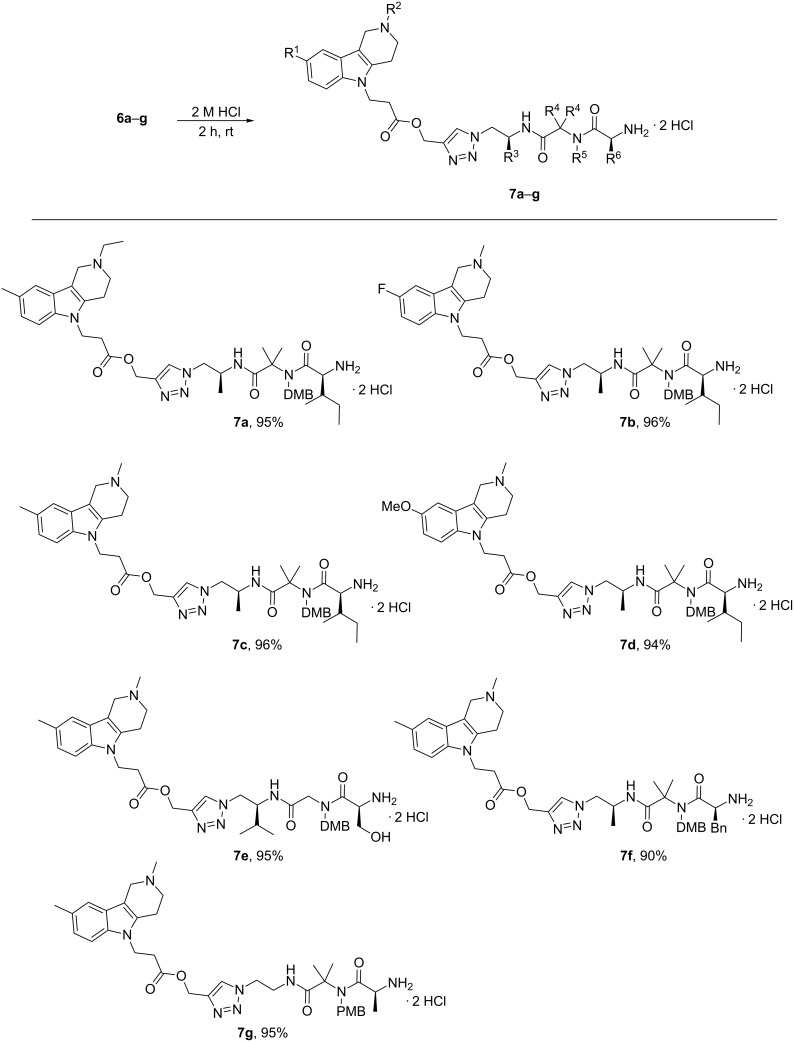
Synthesis of dihydrochloride salts **7a–g**.

Next, we turned our attention to the deprotection of the amine function in the peptide residues, in order to obtain water-soluble conjugates. Thus, *N*-Boc protecting groups were removed from compounds **6a–g** with 2 M HCl in methanol to give the corresponding dihydrochloride salts **7a–g** in nearly quantitative yields (Scheme 4).

The final dihydrochloride salts **7a–g** were tested on rat liver mitochondria (RLM) using standards tests: lipid peroxidation (LP), mitochondrial membrane potential (ΔΨ_m_) and Ca^2+^-induced mitochondrial permeability transition (MPT).

On the day of the experiment, adult male Wistar rats fasted overnight were euthanized in a CO_2_ chamber followed by decapitation. The procedure is in compliance with the Guidelines for Animal Experiments at IPAC RAS. Rat liver mitochondria were isolated by conventional differential centrifugation as previously described [23]. All experiments were provided with mitochondria energized by succinate in the presence of rotenone.

The influence of compounds on spontaneous or induced by *tert*-butylhydroperoxide (*t*-BHP) LP was studied by the standard assay [24]. The extent of LP was measured spectrophotometrically (λ_max_ = 532 nm) and malondialdehyde–thiobarbituric acid adduct concentrations (MDA, expressed in nmol/mg protein) were obtained by interpolation with a MDA standard curve from commercially available 1,1,3,3-tetramethoxypropane. All experiments were repeated using three different preparations of isolated rat liver mitochondria.

Mitochondrial membrane potential was monitored using safranine O [23]. The ability of the compounds to affect MPT was assessed by monitoring mitochondria swelling caused by calcium chloride addition in a plate reader (Victor3, Perkin Elmer). Swelling rate (*V*_max_) was calculated as a slope of the steepest portion of the plot of swelling (light scattering) versus time (dA_530_ × 1000 × min^−1^).

N-substituted tetrahydro-γ-carbolines **3a–d** possessed a pro-oxidant activity. These compounds potentiated the *t*-BHP-induced LP and also induced LP of liver mitochondria. Their peptide conjugates not only lost pro-oxidant activity but, moreover, some of these compounds could inhibit both mitochondrial lipid auto-oxidation and the *t*-BHP-induced LP (Table 1).

**Table 1:** The influence of N-substituted tetrahydro-γ-carbolines and their peptide conjugates on LP of rat liver mitochondria.

Compound	LP in the presence of 0.1 mM compound, nmol MDA/mg protein	Influence of 0.1 mM compound on 1.6 mM *t*-BHP-induced LP, nmol MDA/mg protein

no compounds	0.48 ± 0.01	0.91 ± 0.02
**3a**	1.72 ± 0.05	7.63 ± 0.05
**3b**	1.96 ± 0.04	6.09 ± 0.08
**3c**	1.72 ± 0.08	6.05 ± 0.16
**3d**	1.81 ± 0.08	7.64 ± 0.09
**7a**	0.39 ± 0.02	0.86 ± 0.04
**7b**	0.41 ± 0.02	0.87 ± 0.06
**7c**	0.39 ± 0.02	0.79 ± 0.01
**7d**	0.38 ± 0.02	0.81 ± 0.02
**7e**	0.4 ± 0.03	0.92 ± 0.04
**7f**	0.37 ± 0.01	0.75 ± 0.02
**7g**	0.62 ± 0.02	0.95 ± 0.05

At the concentration of 100 µM (200 nmol/mg mitochondria) the studied compounds caused a week decrease of ΔΨ_m_ and demonstrated no significant influence on mitochondrial swelling (data not shown). But at higher pharmacologically relevant concentration of 30 µM (60 nmol/mg mitochondria) all compounds did not affect the ΔΨ_m_ and increased the resistance of mitochondria to calcium-induced MPT (Table 2). The results of biological evaluation revealed an antioxidant and mitoprotective potential of the new synthesized peptide-modified N-substituted tetrahydro-γ-carbolines. Further investigations of this class of compounds may allow finding a promising approach to cytoprotection, in particularly, neuroprotection.

**Table 2:** The influence of the peptide conjugates of N-substituted tetrahydro-γ-carbolines on rat liver mitochondria swelling.^a^

Compounds	ΔA530/min (% of control)

**7a**	70.5 ± 4.6
**7b**	79.9 ± 8.1
**7c**	80.9 ± 8.0
**7d**	76.2 ± 7.5
**7e**	72.4 ± 2.7
**7f**	70.2 ± 8.6
**7g**	79.0 ± 6.7

^a^Quantification of swelling was measured as the maximal velocity of A530 change after CaCl_2_ addition. The values were normalized against the control values, set to 100%. Data are expressed as means ± SD (*n* = 3 or 4).

## Conclusion

In summary, we described the conjugation of N-substituted tetrahydro-γ-carbolines containing a terminal alkyne group **3a–d** with various azidopeptides **5** (prepared by Ugi multicomponent reaction) through the Cu(I)-catalyzed Huisgen 1,3-dipolar cycloaddition. The activity of the obtained compounds on rat liver mitochondria functional characteristics, such as mitochondrial transmembrane potential, calcium-induced mitochondrial permeability transition and lipid peroxidation of mitochondrial membrane was evaluated. It was found that all compounds at a concentration of 30 µM did not induce depolarization of mitochondria but possessed some inhibitory effect on the mitochondria permeability transition. The starting N-substituted tetrahydro-γ-carbolines **3a–d** demonstrated a high pro-oxidant activity, whereas their peptide conjugates inhibited both auto-oxidation and the *t*-BHP-induced lipid peroxidation.

## Experimental

### General Information

^1^H and ^13^C NMR spectra were recorded in deuterated solvents on a Bruker Avance 400 MHz spectrometer. ^19^F NMR spectra were recorded on a Bruker DXP 200 MHz spectrometer. ^1^H and ^13^C chemical shifts are reported in parts per million (ppm) or δ values downfield from TMS as internal standard. Deuterated solvent peaks were used as internal references: CDCl_3_ at 7.25 and 77.00 ppm. ^19^F chemical shifts are reported on δ scale (in ppm) downfield from CF_3_COOH. Liquid chromatography was performed using Fluka silica gel 60 (0.063–0.200 mm). Melting points were determined with an Electrothermal IA9100 Digital Melting Point Apparatus and are uncorrected. Optical rotations were measured on a Perkin-Elmer 341 polarimeter at 589 nm. High-resolution mass spectra (HRMS) were measured on a MicrOTOF II (Bruker Daltonics) spectrometer.

Compounds **1a–d** were obtained from the respective arylhydrazine hydrochlorides and N-substituted 4-piperidones using Fischer indole synthesis [12–13]. Propargyl acrylate (**2**) was obtained from propargyl alcohol and acryloyl chloride according to procedure described in [25].

**General procedure for the synthesis of compounds 3a–d:** A mixture of correspondingly substituted 2,3,4,5-tetrahydro-1*H*-pyrido[4,3-*b*]indole **1a–d** (2 mmol), 0.22 g (2 mmol) acrylic acid propyn-2-yl ester (**2**) and 0.1 g (0.66 mmol) of CsF in 1 mL of DMF was stirred at 110 °C during 2 h. The solvent was removed in vacuo (~3 mmHg) and the product was extracted from the residue with CH_2_Cl_2_. The solvent was removed in vacuo and the residue was purified by column chromatography (MeOH/CHCl_3_ 1:5).

**General procedure for the Ugi-4CC synthesis of peptides 5:** As described in [14], the corresponding amine (1 mmol) and acetone or CH_2_O (40% in H_2_O, 1 mmol) were dissolved in 5 mL of MeOH and *N*-Boc-protected amino acid (1 mmol) and isocyanide **4** (1 mmol) were added at room temperature. The mixture was stirred for 24 h. The solvent was removed in vacuo and the residue was purified by column chromatography (hexanes/ethyl acetate) to give compounds **5**.

**General procedure for the synthesis of compounds 6a–g:** To a solution of acetylene **3** (0.5 mmol) in 5 mL of CH_2_Cl_2_ was added the peptide **5** (0.5 mmol), 0.012 g (0.05 mmol) of CuSO_4_·5H_2_O in 0.25 mL of H_2_O and 0.04 g (0.2 mmol) of sodium ascorbate in 0.25 mL of H_2_O. The reaction mixture was stirred at 40 °С for 1 h. After the reaction was completed 10 mL of CH_2_Cl_2_ was added and the reaction mixture was washed with aq NH_3_ and then with water. The organic layer was separated and dried over Na_2_SO_4_. The solvent was removed in vacuo and the residue was purified by column chromatography (CH_2_Cl_2_/MeOH 10:1).

**General procedure for the synthesis of dihydrochlorides 7a–g:** The corresponding compound **6a–g** (0.12 mmol) was dissolved in 1 mL (2 mmol) of a 2 M solution of HCl in MeOH. The reaction mixture was stirred at room temperature for 2 h. The solvent was removed in vacuo and the residue was dissolved in 5 mL of EtOH. Then the solvent was evaporated and 5 mL of acetonitrile were added to the residue. After evaporation of the solvent the corresponding dihydrochlorides **7a–g** were obtained.

## Supporting Information

File 1 General information and characterization data for all compounds.
