# Supplementary material for: Synthesis and biological activity of N-substituted-tetrahydro-γ-carbolines containing peptide residues
Source: Beilstein J Org Chem. 2014 Jan 15;10:155–62. doi: 10.3762/bjoc.10.13 (PMC3896248; doi:10.3762/bjoc.10.13)
Supplement: File 1 — General information and characterization data for all compounds. [file Beilstein_J_Org_Chem-10-155-s001.pdf]

Supporting information

for

Synthesis and biological activity of *N*-substituted-tetrahydro-γ-carbolines containing peptide residues

Nadezhda V. Sokolova[1,2], Valentine G. Nenajdenko*[1,3,§], Vladimir B. Sokolov[2], Daria V. Vinogradova[2], Elena F. Shevtsova[2], Ludmila G. Dubova[2] and Sergey O. Bachurin*[2]

Address: [1]Department of Chemistry, Moscow State University, Leninskie Gory 1, Moscow, 119992, Russia, [2]Institute of Physiologically Active Compounds Russian Academy of Sciences, Severny proezd 1, Chernogolovka, 142432, Russia and [3]A. N. Nesmeyanov Institute of Organoelement Compounds, Russian Academy of Sciences, Vavilov str. 28, 119991 Moscow, Russian Federation

Email: Valentine G. Nenajdenko - nen@acylium.chem.msu.ru; Sergey O. Bachuring - bachurin@ipac.ac.ru

[§]Phone: +7 495 9392276; Fax: +7 495 9328846

*Corresponding author

General information and characterization data for all compounds.

Table of Contents

Characterization of compounds **3a–d** S2
Characterization of peptides **5** S3
Characterization of compounds **6a–g** S4
Characterization of compounds **7a–g** S7

Characterization of compounds **3a**–**d**

3-(2-Ethyl-8-methyl-1,2,3,4-tetrahydro-5*H*-pyrido[4,3-*b*]indol-5-yl)-propionic acid prop-2-ynyl ester (3a): According to the general procedure, **3a** was obtained from 2-ethyl-8-methyl-2,3,4,5-tetrahydro-1*H*-pyrido[4,3-*b*]indole **1a** as a yellow oil in 77 % yield; R_f (MeOH / $CDCl_3$ 1:5) 0.7; ^{1}HNMR (400 MHz, $CDCl_3$) δ 1.23 (t, *J* = 6.2 Hz, 3H, CH_3), 2.42 (s, 3H, CH_3), 2.45-2.47 (m, 1H, CH), 2.69 (q, *J* = 7.1 Hz, 2H, $C\underline{H_2}CH_3$), 2.76 (t, *J* = 7.2 Hz, 2H, CH_2), 2.82-2.92 (m, 4H, 2 CH_2), 3.68 (s, 2H, CH_2), 4.31 (t, *J* = 7.0 Hz, 2H, CH_2), 4.64 (d, *J* = 2.4 Hz, 2H, CH_2), 6.97 (d, *J* = 7.3 Hz, 1H, Ar), 7.13-7.20 (m, 2H, Ar); ^{13}CNMR (100 MHz, $CDCl_3$) δ 12.1, 21.3, 22.1, 34.3, 38.3, 48.8, 50.1, 51.3, 52.1, 75.1, 77.2, 106.7, 108.4, 117.5, 122.5, 126.1, 128.4, 132.7, 134.4, 170.2; Anal. Calcd for $C_{20}H_{24}N_2O_2$ (324.4168): C, 74.05; H, 7.46; found: C, 74.18; H, 7.62.

3-(8-Fluoro-2-methyl-1,2,3,4-tetrahydro-5*H*-pyrido[4,3-*b*]indol-5-yl)-propionic acid prop-2-ynyl ester (**3b**): According to the general procedure, **3b** was obtained from 8-fluoro-2-methyl-2,3,4,5-tetrahydro-1*H*-pyrido[4,3-*b*]indole **1b** as a yellow oil in 81 % yield; R_f (MeOH / $CDCl_3$ 1:5) 0.65; ^{1}HNMR (400 MHz, $CDCl_3$) δ 2.44-2.47 (m, 1H, CH), 2.57 (s, 3H, CH_3), 2.76 (t, *J* = 7.2 Hz, 2H, CH_2), 2.84-2.92 (m, 4H, 2 CH_2), 3.64 (s, 2H, CH_2), 4.32 (t, *J* = 7.1 Hz, 2H, CH_2), 4.64 (d, *J* = 2.4 Hz, 2H, CH_2), 6.88 (td, *J* = 9.3 Hz, *J* = 2.4 Hz, 1H, Ar), 7.03 (dd, *J* = 7.1 Hz, *J* = 2.3 Hz, 1H, Ar), 7.17 (dd, *J* = 9.1 Hz, *J* = 4.4 Hz, 1H, Ar); ^{13}C NMR (100 MHz, $CDCl_3$) δ 22.3, 34.0, 38.2, 45.0, 51.1, 51.8, 51.9, 75.1, 77.2, 102.5 (d, $^2J_{C\text{-}F}$ = 22.8 Hz), 107.7, 108.5 (d, $^2J_{C\text{-}F}$ = 25.8 Hz), 109.1, 125.7, 132.3, 134.4, 157.4 (d, $^1J_{C\text{-}F}$ = 233.3 Hz), 169.9; ^{19}F (188 MHz, $CDCl_3$) δ -47.4 (td, *J* = 9.4 Hz, *J* = 4.3 Hz); Anal. Calcd for $C_{18}H_{19}FN_2O_2$ (314.3541): C, 68.77; H, 6.09; found: C, 68.92; H, 6.17.

3-(2,8-Dimethyl-1,2,3,4-tetrahydro-5*H*-pyrido[4,3-*b*]indol-5-yl)-propionic acid prop-2-ynyl ester (**3c**): According to the general procedure, **3c** was obtained from 2.8-dimethyl-2,3,4,5-tetrahydro-1*H*-pyrido[4,3-*b*]indole **1c** as a yellow oil in 72 % yield; R_f (MeOH / $CDCl_3$ 1:5) 0.6; ^{1}HNMR (400 MHz, $CDCl_3$) δ 2.42 (s, 3H, CH_3), 2.46 (t, *J* = 2.4 Hz, 1H, CH), 2.55 (s, 3H, CH_3), 2.76 (t, *J* = 7.2 Hz, 2H, CH_2), 2.85-2.90 (m, 4H, 2 CH_2), 3.67 (s, 2H, CH_2), 4.32 (t, *J* = 7.1 Hz, 2H, CH_2), 4.64 (d, *J* = 2.4 Hz, 2H, CH_2), 6.98 (d, *J* = 7.3 Hz, 1H, Ar), 7.14-7.19 (m, 2H, Ar); ^{13}C NMR (100 MHz, $CDCl_3$) δ 21.3, 22.2, 34.3, 38.4, 45.0, 51.4, 52.1, 52.2, 75.2, 77.2, 107.3, 108.5, 117.6, 122.5, 126.1, 128.5, 132.5, 134.4, 170.3; Anal. Calcd for $C_{19}H_{22}N_2O_2$ (310.3903): C, 73.52; H, 7.14; found: C, 73.69; H, 7.33.

3-(8-Methoxy-2-methyl-1,2,3,4-tetrahydro-5*H*-pyrido[4,3-*b*]indol-5-yl)-propionic acid prop-2-ynyl ester (**3d**): According to the general procedure, **3d** was obtained from 8-methoxy-2-methyl-2,3,4,5-tetrahydro-1*H*-pyrido[4,3-*b*]indole **1d** as a yellow oil in 75 % yield; R_f (MeOH / $CDCl_3$ 1:5) 0.5; ^{1}HNMR (400 MHz, $CDCl_3$) δ 2.47 (t, *J* = 2.4 Hz, 1H, CH), 2.57 (s, 3H, CH_3), 2.74 (t, *J* = 7.1 Hz, 2H, CH_2), 2.89 (br. s, 4H, 2 CH_2), 3.68 (s, 2H, CH_2), 3.81 (s, 3H, OCH_3), 4.29 (t, *J* = 7.1 Hz, 2H, CH_2), 4.62 (d, *J* = 2.4 Hz, 2H, CH_2), 6.78 (dd, *J* = 8.8 Hz, *J* = 2.4 Hz, 1H, Ar), 6.81-6.85 (m, 1H, Ar), 7.15 (d, *J* = 8.8 Hz, 1H, Ar); ^{13}C NMR (100 MHz, $CDCl_3$) δ 22.4, 34.3, 38.4, 45.2, 51.5, 52.2, 55.6, 55.8, 75.2, 77.2, 100.1, 107.3, 109.4, 110.6, 126.0, 131.1, 133.1, 153.9, 170.3; Anal. Calcd for $C_{19}H_{22}N_2O_3$ (326.3897): C, 69.92; H, 6.79; found: C, 69.76; H, 6.84.

Characterization of peptides **5**

***N*-(*tert*-Butoxycarbonyl)-L-seryl-*N*^{1}-[(1*S*)-1-(azidomethyl)-2-methylpropyl]-*N*^{2}-(2,4-dimethoxybenzyl)glycinamide (mixture of rotamers, ~ 4:1) (5b):** According to the general procedure for Ugi-4CC, **5b** was obtained from 2,4-dimethoxybenzylamine, CH_2O, *N*-Boc-*L*-serine and (*S*)-1-azido-2-isocyano-3-methylbutane. Yield 65%; white solid; mp 57-58 °C; R_f (ethyl acetate) 0.7; $[\alpha]_D^{20}$ -31.9 (*c* 0.7, CH_2Cl_2); ^{1}H NMR ($CDCl_3$, 400 MHz) δ 0.85 (d, *J* = 6.7 Hz, 3H, CH_3), 0.90 (d, *J* = 6.7 Hz, 3H, CH_3), 1.43 (s, 9H, 3 CH_3-Boc), 1.73-1.81 (m, 1H, CH), 3.38 (d, *J* = 4.9 Hz, 2H, CH_2), 3.54-3.59 (m, 1H, CH_2), 3.65-3.70 (m, 1H, CH_2), 3.73 (s, 3H, OCH_3), 3.77 (s, 3H, OCH_3), 3.78-3.84 (m, 1H, CH_2), 3.89-3.96 (m, 1H, CH_2), 4.11-4.22 (m, 1H, CH), 4.32 (d, J_{AB} = 15.5 Hz, 1H, CH_2), 4.82 (d, J_{AB} = 15.5 Hz, 1H, CH_2), 5.13-5.22 (m, 1H, CH), 5.57 (d, *J* = 7.3 Hz, 0.20*1H, NH), 5.66 (d, *J* = 7.3 Гц, 0.80*1H, NH), 6.35-6.42 (m, 2H, Ar), 6.50 (d, *J* = 9.0 Hz, 0.80*1H, NH), 6.90 (d, *J* = 9.0 Hz, 0.20*1H, NH), 7.01 (d, *J* = 8.6 Hz, 0.80*1H, Ar), 7.13 (d, *J* = 8.6 Hz, 0.20*1H, Ar); ^{13}C NMR (100 MHz, $CDCl_3$) δ 18.7, 19.3, 28.2, 29.4, 48.5, 51.4, 52.5, 54.3, 55.0, 55.3, 64.6, 79.9, 98.8, 103.9, 115.4, 130.9, 155.5, 158.7, 161.2, 168.5, 171.9; Anal. Calcd for $C_{24}H_{38}N_6O_7$ (522.5948): C, 55.16; H, 7.33; N, 16.08; found: C, 55.24; H, 7.35; N, 16.10.

The synthesis and full characterization of peptides **5a,c,d** can be found in [14] and [15].

Characterization of compounds **6a**–**g**

***N*-(*tert*-Butoxycarbonyl)-L-isoleucyl-*N*^{2}-(2,4-dimethoxybenzyl)-*N*^{1}-{(1*S*)-2-[4-({[3-(2-ethyl-8-methyl-1,2,3,4-tetrahydro-5*H*-pyrido[4,3-*b*]indol-5-yl)propanoyl]oxy}methyl)-1*H*-1,2,3-triazol-1-yl]-1-methylethyl}-2-methylalaninamide (6a)**: Yield 70%; white solid; mp 94 °C; R_f (CH_2Cl_2 / MeOH 10:1) 0.5; $[\alpha]_D^{20}$ -25.3 (*c* 0.5, CH_2Cl_2); ^{1}H NMR ($CDCl_3$, 400 MHz) δ 0.78-0.88 (m, 6H, 2 CH_3), 0.96-1.04 (m, 1H, CH), 1.12 (d, *J* = 6.3 Hz, 3H, CH_3), 1.21-1.27 (m, 3H, CH_3), 1.35 (br. s, 6H, 2 CH_3), 1.39 (s, 9H, 3 CH_3-Boc), 1.44-1.55 (m, 1H, CH_2), 1.64-1.72 (m, 1H, CH_2), 2.41 (s, 3H, CH_3), 2.74 (t, *J* = 7.2 Hz, 2H, CH_2), 2.86-3.05 (m, 4H, 2 CH_2), 3.09-3.20 (m, 2H, CH_2), 3.77 (s, 6H, 2 OCH_3), 3.89-3.97 (m, 2H, CH_2), 4.17-4.25 (m, 1H, CH), 4.29 (t, *J* = 7.1 Hz, 2H, CH_2), 4.36-4.45 (m, 2H, CH_2, 1H, CH), 4.52-4.62 (m, 2H, CH_2), 5.11-5.18 (m, 2H, CH_2, 1H, NH), 5.73-5.79 (m, 1H, NH), 6.40-6.48 (m, 2H, Ar), 6.97 (d, *J* = 7.3 Hz, 1H, Ar), 7.11-7.17 (m, 2H, Ar), 7.31-7.38 (m, 1H, Ar), 7.60 (s, 1H, CH-triazole); ^{13}C NMR (100 MHz, $CDCl_3$) δ 11.4, 12.2, 15.7, 17.5, 21.3, 22.3, 23.4, 23.7, 28.3, 34.5, 38.6, 38.7, 42.5, 45.6, 48.9, 50.2, 51.5, 53.9, 55.3, 55.8, 57.9, 62.9, 79.3, 98.7, 104.3, 108.6, 117.5, 118.2, 122.6, 124.9, 126.1, 128.4, 128.9, 132.8, 134.5, 142.2, 155.3, 157.3, 160.4, 170.9, 173.6, 174.7; HRMS (ESI) calculated for $C_{47}H_{68}N_8O_8$ $[M + H]^+$ 873.5238, found 873.5236.

***N*-(*tert*-Butoxycarbonyl)-L-isoleucyl-*N*^{2}-(2,4-dimethoxybenzyl)-*N*^{1}-{(1*S*)-2-[4-({[3-(8-fluoro-2-methyl-1,2,3,4-tetrahydro-5*H*-pyrido[4,3-*b*]indol-5-yl)propanoyl]oxy}methyl)-1*H*-1,2,3-triazol-1-yl]-1-methylethyl}-2-methylalaninamide (6b)**: Yield 60%; white solid; mp 101 °C; R_f (CH_2Cl_2 / MeOH 10:1) 0.35; $[\alpha]_D^{20}$ -24.9 (*c* 0.5, CH_2Cl_2); ^{1}H NMR ($CDCl_3$, 400 MHz) δ 0.78-0.90 (m, 6H, 2 CH_3), 0.95-1.06 (m, 1H, CH), 1.12 (d, *J* = 6.3 Hz, 3H, CH_3), 1.25 (br. s, 3H, CH_3), 1.39 (br. s, 9H, 3 CH_3-Boc, 3H, CH_3), 1.44-1.57 (m, 1H, CH_2), 1.62-1.72 (m, 1H, CH_2), 2.59 (s, 3H, CH_3), 2.72 (t, *J* = 7.2 Hz, 2H, CH_2), 2.92 (br. s, 4H, 2 CH_2), 3.68 (br. s, 2H, CH_2), 3.77 (s, 6H, 2 OCH_3), 4.20-4.26 (m, 1H, CH), 4.30 (t, *J* = 7.1 Hz, 2H, CH_2), 4.37-4.48 (m, 2H, CH_2, 1H, CH), 4.52-4.64 (m, 2H, CH_2), 5.10-5.19 (m, 2H, CH_2, 1H, NH), 5.74-5.83 (m, 1H, NH), 6.39-6.48 (m, 2H, Ar), 6.85 (td, *J* = 9.6 Hz, *J* = 2.0 Hz, 1H, Ar), 6.97 (d, *J* = 7.2 Hz, 1H, Ar), 7.15 (dd, *J* = 6.9 Hz, *J* = 4.2 Hz, 1H, Ar), 7.31-7.40 (m, 1H, Ar), 7.60 (s, 1H, CH-triazole); ^{13}C NMR (100 MHz, $CDCl_3$) δ 11.3, 15.6, 17.5, 22.6, 23.4, 23.7, 28.2, 34.5, 38.7, 42.5, 45.3, 45.7, 51.4, 52.2, 53.9, 55.3, 55.7, 57.9, 62.9, 79.3, 98.7, 102.7 (d, $^2J_{C\text{-}F}$ = 23.6 Hz), 104.3, 108.9 (d, $^2J_{C\text{-}F}$ = 26.2 Hz), 109.3, 109.4, 118.2, 124.9, 125.9, 128.9, 132.6, 134.5, 142.0, 155.3, 157.3, 157.6 (d, $^1J_{C\text{-}F}$ = 233.3 Hz), 160.4, 170.6, 173.6, 174.7; ^{19}F (188 MHz, $CDCl_3$) δ -47.22 (td, *J* =

9.4 Hz, J = 4.3 Hz, F, Ar); HRMS (ESI) calculated for $C_{45}H_{63}FN_8O_8$ $[M + H]^+$ 863.4831, found 863.4822.

***N*-(*tert*-Butoxycarbonyl)-L-isoleucyl-*N*²-(2,4-dimethoxybenzyl)-*N*¹-{(1*S*)-2-[4-({[3-(2,8-dimethyl-1,2,3,4-tetrahydro-5*H*-pyrido[4,3-*b*]indol-5-yl)propanoyl]oxy}methyl)-1*H*-1,2,3-triazol-1-yl]-1-methylethyl}-2-methylalaninamide (6c)**: Yield 68%; white solid; mp 105 °C; R_f (CH_2Cl_2 / MeOH 10:1) 0.45; $[\alpha]_D^{20}$ -25.0 (*c* 0.4, CH_2Cl_2); ^{1}H NMR ($CDCl_3$, 400 MHz) δ 0.82 (t, J = 7.4 Hz, 3H, CH_3), 0.86 (d, J = 6.8 Hz, 3H, CH_3), 0.96-1.05 (m, 1H, CH), 1.12 (d, J = 6.6 Hz, 3H, CH_3), 1.24 (br. s, 3H, CH_3), 1.36 (br. s, 3H, CH_3), 1.39 (br. s, 9H, 3 CH_3-Boc), 1.45-1.55 (m, 1H, CH_2), 1.62-1.72 (m, 1H, CH_2), 2.41 (s, 3H, CH_3), 2.67 (s, 3H, CH_3), 2.74 (t, J = 6.9 Hz, 2H, CH_2), 2.94-3.08 (m, 4H, 2 CH_2), 3.78 (s, 6H, 2 OCH_3), 3.81-3.88 (m, 2H, CH_2), 4.20-4.26 (m, 1H, CH), 4.30 (t, J = 7.1 Hz, 2H, CH_2), 4.37-4.46 (m, 2H, CH_2, 1H, CH), 4.51-4.62 (m, 2H, CH_2), 5.10-5.19 (m, 2H, CH_2, 1H, NH), 5.75 (d, J = 7.0 Hz, 1H, NH), 6.40-6.48 (m, 2H, Ar), 6.97 (d, J = 7.3 Hz, 1H, Ar), 7.13-7.18 (m, 2H, Ar), 7.31-7.38 (m, 1H, Ar), 7.58 (s, 1H, CH-triazole); ^{13}C NMR (100 MHz, $CDCl_3$) δ 11.3, 15.6, 17.4, 21.3, 21.6, 23.3, 23.6, 28.2, 34.3, 38.5, 42.5, 44.4, 45.6, 51.3, 51.9, 53.3, 53.8, 55.2, 55.7, 57.9, 62.8, 79.2, 98.6, 104.2, 105.6, 108.6, 117.4, 118.2, 122.8, 124.9, 125.7, 128.6, 128.8, 131.9, 134.4, 142.0, 155.3, 157.3, 160.3, 170.7, 173.5, 174.6; HRMS (ESI) calculated for $C_{46}H_{66}N_8O_8$ $[M + H]^+$ 859.5082, found 859.5041.

***N*-(*tert*-Butoxycarbonyl)-L-isoleucyl-*N*²-(2,4-dimethoxybenzyl)-*N*¹-{(1*S*)-2-[4-({[3-(8-methoxy-2-methyl-1,2,3,4-tetrahydro-5*H*-pyrido[4,3-*b*]indol-5-yl)propanoyl]oxy}methyl)-1*H*-1,2,3-triazol-1-yl]-1-methylethyl}-2-methylalaninamide (6d)**: Yield 65%; white solid; mp 101-103 °C; R_f (CH_2Cl_2 / MeOH 10:1) 0.35; $[\alpha]_D^{20}$ -23.0 (*c* 0.7, CH_2Cl_2); ^{1}H NMR ($CDCl_3$, 400 MHz) δ 0.79-0.88 (m, 6H, 2 CH_3), 0.95-1.04 (m, 1H, CH), 1.12 (d, J = 6.7 Hz, 3H, CH_3), 1.24 (br. s, 3H, CH_3), 1.36 (br. s, 3H, CH_3), 1.38 (br. s, 9H, 3 CH_3-Boc), 1.44-1.55 (m, 1H, CH_2), 1.62-1.72 (m, 1H, CH_2), 2.58 (s, 3H, CH_3), 2.70 (t, J = 7.0 Hz, 2H, CH_2), 2.89 (br. s, 4H, 2 CH_2), 3.69 (br. s, 2H, CH_2), 3.77 (br. s, 6H, 2 OCH_3), 3.81 (s, 3H, OCH_3), 4.19-4.25 (m, 1H, CH), 4.27 (t, J = 7.0 Hz, 2H, CH_2), 4.36-4.47 (m, 2H, CH_2, 1H, CH), 4.51-4.60 (m, 2H, CH_2), 5.10-5.17 (m, 2H, CH_2, 1H, NH), 5.74-5.84 (m, 1H, NH), 6.40-6.47 (m, 2H, Ar), 6.77 (dd, J = 8.8 Hz, J = 2.4 Hz, 1H, Ar), 6.82 (d, J = 2.4 Hz, 1H, Ar), 7.14 (d, J = 8.8 Hz, 1H, Ar), 7.31-7.36 (m, 1H, Ar), 7.56 (s, 1H, CH-triazole); ^{13}C NMR (100 MHz, $CDCl_3$) δ 11.4, 15.6, 17.5, 22.4, 23.4, 23.7, 28.2, 30.9, 34.6, 38.6, 42.5, 45.2, 45.6, 51.6, 52.2, 53.8, 55.3, 55.7, 55.8, 55.9, 57.9, 62.9, 79.3, 98.6, 100.2, 104.3, 107.1, 109.6, 110.6, 118.2, 124.9, 126.0, 128.9, 131.2, 133.2, 142.1, 153.9,

155.3, 157.3, 160.3, 170.8, 173.6, 174.7; HRMS (ESI) calculated for $C_{46}H_{66}N_8O_9$ $[M + H]^+$ 875.5031, found 875.5020.

***N*-(*tert*-Butoxycarbonyl)-L-seryl-N^2-(2,4-dimethoxybenzyl)-N^1-((1*S*)-1-{[4-({[3-(2,8-dimethyl-1,2,3,4-tetrahydro-5*H*-pyrido[4,3-*b*]indol-5-yl)propanoyl]oxy}methyl)-1*H*-1,2,3-triazol-1-yl]methyl}-2-methylpropyl)glycinamide (mixture of rotamers, ~ 4:1) (6e)**: Yield 65%; white solid; mp 113 °C; R_f (CH_2Cl_2 / MeOH 10:1) 0.55; $[\alpha]_D^{20}$ -9.4 (*c* 0.5, CH_2Cl_2); ^1H NMR ($CDCl_3$, 400 MHz) δ 0.94 (d, *J* = 6.8 Hz, 3H, CH_3), 1.04 (d, *J* = 6.8 Hz, 3H, CH_3), 1.42 (s, 9H, 3 CH_3-Boc), 1.68-1.76 (m, 1H, CH), 2.41 (s, 3H, CH_3), 2.60 (s, 3H, CH_3), 2.79 (t, *J* = 6.8 Hz, 2H, CH_2), 2.83-2.97 (m, 4H, 2 CH_2), 3.50-3.61 (m, 2H, CH_2), 3.68-3.73 (m, 1H, CH_2), 3.74 (s, 3H, OCH_3), 3.75-3.78 (m, 1H, CH_2), 3.79 (s, 3H, OCH_3), 3.82-3.88 (m, 1H), 3.90-3.99 (m, 1H), 4.12 (d, J_{AB} = 15.3 Hz, 1H, CH_2), 4.24 (d, J_{AB} = 15.3 Hz, 1H, CH_2), 4.29-4.48 (m, 5H), 4.80 (d, J_{AB} = 15.1 Hz, 1H, CH_2), 5.15 (d, J_{AB} = 12.7 Hz, 1H, CH_2), 5.25 (d, J_{AB} = 12.7 Hz, 1H, CH_2), 5.52 (d, *J* = 7.1 Hz, 0.20*1H, NH), 5.82 (d, *J* = 7.1 Hz, 0.80*1H, NH), 6.38-6.45 (m, 2H, Ar), 6.95 (d, *J* = 8.7 Hz, 1H, Ar), 7.01 (d, *J* = 8.6 Hz, 1H, Ar), 7.12-7.17 (m, 2H, Ar), 7.48 (s, 1H, CH-triazole); ^{13}C NMR (100 MHz, $CDCl_3$) δ 18.7, 19.3, 21.3, 21.9, 28.2, 29.5, 34.4, 38.5, 44.9, 48.1, 50.7, 51.5, 52.2, 55.0, 55.3, 57.9, 64.2, 79.6, 98.1, 98.7, 103.9, 104.4, 106.1, 108.6, 115.2, 117.3, 122.7, 124.9, 125.6, 128.5, 130.9, 132.2, 134.4, 142.1, 155.2, 158.7, 161.2, 168.5, 170.9, 171.8; HRMS (ESI) calculated for $C_{43}H_{60}N_8O_9$ $[M + H]^+$ 833.4561, found 833.4548.

***N*-(*tert*-Butoxycarbonyl)-L-phenylalanyl-N^2-(2,4-dimethoxybenzyl)-N^1-{(1*S*)-2-[4-({[3-(2,8-dimethyl-1,2,3,4-tetrahydro-5*H*-pyrido[4,3-*b*]indol-5-yl)propanoyl]oxy}methyl)-1*H*-1,2,3-triazol-1-yl]-1-methylethyl}-2-methylalaninamide (6f)**: Yield 67%; white solid; mp 103 °C; R_f (CH_2Cl_2 / MeOH 10:1) 0.4; $[\alpha]_D^{20}$ -11.5 (*c* 0.5, CH_2Cl_2); ^1H NMR ($CDCl_3$, 400 MHz) δ 1.13 (d, *J* = 6.8 Hz, 3H, CH_3), 1.21 (br. s, 3H, CH_3), 1.33 (br. s, 3H, CH_3, 9H, 3 CH_3-Boc), 2.40 (s, 3H, CH_3), 2.57 (s, 3H, CH_3), 2.72 (t, *J* = 6.8 Hz, 2H, CH_2), 2.78-3.00 (m, 6H, 3 CH_2), 3.66-3.71 (m, 2H, CH_2), 3.73 (s, 3H, OCH_3), 3.76 3 (s, 3H, OCH_3), 4.19-4.33 (m, 4H), 4.36-4.51 (m, 2H), 4.59-4.66 (m, 1H), 5.13-5.20 (m, 3H), 5.83 (d, *J* = 7.1 Hz, 1H, NH), 6.38-6.44 (m, 2H, Ar), 6.95 (d, *J* = 8.2 Hz, 1H, Ar), 7.04-7.09 (m, 2H, Ar), 7.11-7.16 (m, 2H, Ar), 7.18-7.24 (m, 4H, Ar), 7.69 (s, 1H, CH-triazole); ^{13}C NMR (100 MHz, $CDCl_3$) δ 17.3, 21.2, 22.1, 23.0, 23.8, 28.1, 34.3, 38.4, 39.6, 42.2, 45.0, 45.4, 51.4, 52.0, 52.8, 53.3, 53.6, 55.1, 55.2, 57.8, 62.6, 79.4, 98.4, 104.1, 106.7, 108.5, 117.3, 118.0, 122.4, 125.0, 125.8, 126.6, 128.2, 128.3, 129.4, 132.4,

134.2, 136.4, 142.0, 154.7, 157.0, 160.0, 170.7, 172.9, 174.4; HRMS (ESI) calculated for $C_{49}H_{64}N_8O_8$ $[M + H]^+$ 893.4925, found 893.4903.

***N*-(*tert*-Butoxycarbonyl)-L-alanyl-N^1-{2-[4-({[3-(2,8-dimethyl-1,2,3,4-tetrahydro-5*H*-pyrido[4,3-*b*]indol-5-yl)propanoyl]oxy}methyl)-1*H*-1,2,3-triazol-1-yl]ethyl}-N^2-(4-methoxybenzyl)-2-methylalaninamide (6g)**: Yield 60%; white solid; mp 88 °C; R_f (CH_2Cl_2 / MeOH 10:1) 0.4; $[\alpha]_D^{20}$ +3.0 (*c* 0.1, CH_2Cl_2); ^1H NMR ($CDCl_3$, 400 MHz) δ 1.12 (d, J = 6.6 Hz, 3H, CH_3), 1.30 (br. s, 3H, CH_3), 1.37 (br. s, 3H, CH_3, 9H, 3 CH_3-Boc), 2.40 (s, 3H, CH_3), 2.52 (s, 3H, CH_3), 2.71 (t, J = 6.9 Hz, 2H, CH_2), 2.75-2.83 (m, 4H, 2 CH_2), 3.56-3.71 (m, 4H, 2 CH_2), 3.78 (s, 3H, OCH_3), 4.30 (t, J = 7.1 Hz, 2H, CH_2), 4.37-4.46 (m, 1H, CH, 2H, CH_2), 4.56 (d, J_{AB} = 17.1 Hz, 1H, CH_2), 4.77 (d, J_{AB} = 17.1 Hz, 1H, CH_2), 5.14 (d, J_{AB} = 12.7 Hz, 1H, CH_2), 5.20 (d, J_{AB} = 12.7 Hz, 1H, CH_2), 5.34 (d, J = 7.8 Hz, 1H, NH), 6.17-6.25 (m, 1H, NH), 6.88 (d, J = 8.7 Hz, 2H, Ar), 6.94 (d, J = 7.3 Hz, 1H, Ar), 7.12-7.17 (m, 2H, Ar), 7.28 (d, J = 8.7 Hz, 2H, Ar), 7.69 (s, 1H, CH-triazole); ^{13}C NMR (100 MHz, $CDCl_3$) δ 18.5, 21.3, 22.8, 24.1, 28.2, 34.6, 38.5, 39.9, 45.6, 47.0, 47.4, 49.2, 51.7, 52.4, 55.2, 57.9, 62.8, 79.7, 107.8, 108.5, 114.3, 117.5, 122.3, 125.2, 126.0, 127.3, 128.3, 129.9, 132.9, 134.4, 142.2, 155.4, 158.9, 170.9, 174.3, 175.2. HRMS (ESI) calculated for $C_{41}H_{56}N_8O_7$ $[M + H]^+$ 773.4350, found 773.4333.

Characterization of compounds **7a**–**g**

L-Isoleucyl-N^2-(2,4-dimethoxybenzyl)-N^1-{(1*S*)-2-[4-({[3-(2-ethyl-8-methyl-1,2,3,4-tetrahydro-5*H*-pyrido[4,3-*b*]indol-5-yl)propanoyl]oxy}methyl)-1*H*-1,2,3-triazol-1-yl]-1-methylethyl}-2-methylalaninamide dihydrochloride (mixture of rotamers) (7a): According to the general procedure **7a** was obtained from **6a** as a white solid in 95% yield; mp 125 °C; $[\alpha]_D^{25}$ +0.9 (*c* 0.3, MeOH); ^1H NMR (DMSO-d_6, 400 MHz) δ 0.75-0.84 (m, 6H, 2 CH_3), 0.93-1.02 (m, 1H, CH), 1.08 (d, J = 6.3 Hz, 3H, CH_3), 1.18 (s, 3H, CH_3), 1.20 (s, 3H, CH_3), 1.36 (t, J = 6.8 Hz, 3H, CH_3), 1.42-1.52 (m, 1H, CH_2), 1.81-1.91 (m, 1H, CH_2), 2.36 (s, 3H, CH_3), 2.63-2.77 (m, 2H, CH_2), 3.08-3.29 (m, 4H, 2 CH_2), 3.34-3.45 (m, 1H, CH_2), 3.51-3.58 (m, 1H, CH_2), 3.74 and 3.75 (s, 6H, 2 OCH_3), 3.81 (br. s, 2H, CH_2), 4.09-4.20 (m, 2H), 4.26-4.39 (м, 3H), 4.44-4.55 (m, 3H), 4.69-4.80 (m, 2H), 5.02-5.07 (m, 1H, NH), 6.50 (d, J = 6.8 Hz, 1H, Ar), 6.58 (br. s, 1H, Ar), 6.97 (d, J = 7.5 Hz, 1H, Ar), 7.23-7.29 (m, 1H, Ar), 7.41-7.54 (m, 2H, Ar), 7.92 (s, 0.60*1H, CH-triazole), 8.07 (s, 0.40*1H, CH-triazole),8.43 and 8.45 (br. s, 2H); ^{13}C NMR (100

MHz, DMSO-d_6) δ 9.3, 10.9, 15.1, 17.5, 21.1, 22.6, 23.3, 23.8, 33.9, 35.7, 42.4, 45.7, 47.1, 48.4, 49.8, 51.6, 52.9, 54.9, 55.2, 55.6, 62.7, 98.3, 104.5, 109.5, 117.5, 117.7, 123.0, 123.2, 124.9, 128.0, 128.8, 131.4, 134.4, 141.1, 156.8, 159.9, 169.1, 171.3, 173.3; Anal. Calcd for $C_{42}H_{62}Cl_2N_8O_6{\cdot}3H_2O$ (899.9432): C, 56.05; H, 7.62; found: C, 55.53; H, 6.93.

L-Isoleucyl-*N*²-(2,4-dimethoxybenzyl)-*N*¹-{(1*S*)-2-[4-({[3-(8-fluoro-2-methyl-1,2,3,4-tetrahydro-5*H*-pyrido[4,3-*b*]indol-5-yl)propanoyl]oxy}methyl)-1*H*-1,2,3-triazol-1-yl]-1-methylethyl}-2-methylalaninamide dihydrochloride (mixture of rotamers) (7b): According to the general procedure **7b** was obtained from **6b** as a white solid in 96% yield; mp 143 °C; $[\alpha]_D^{25}$ +2.0 (*c* 0.1, MeOH); ^{1}H NMR (DMSO-d_6, 400 MHz) δ 0.73-0.86 (m, 6H, 2 CH_3), 0.94-1.02 (m, 1H, CH), 1.07 (d, J = 6.3 Hz, 3H, CH_3), 1.18 (s, 3H, CH_3), 1.21 (s, 3H, CH_3), 1.43-1.51 (m, 1H, CH_2), 1.81-1.90 (m, 1H, CH_2), 2.70-2.79 (m, 2H, CH_2), 2.89 and 2.90 (s, 3H, CH_3), 3.18-3.28 (m, 1H, CH_2), 3.38-3.49 (m, 1H, CH_2), 3.53-3.58 (m, 1H, CH_2), 3.64-3.71 (m, 1H, CH_2), 3.74, 3.75, 3.78 and 3.81 (s, 6H, 2 OCH_3), 4.09-4.24 (m, 2H), 4.31-4.58 (m, 9H), 4.75 (d, J_{AB} = 18.4 Hz, 1H, CH_2), 5.03-5.08 (m, 1H, NH), 6.48-6.58 (m, 2H, Ar), 6.92-7.02 (m, 1H, Ar), 7.22-7.27 (m, 1H, Ar), 7.44-7.52 (m, 2H, Ar), 7.92 (s, 0.60*1H, CH-triazole), 8.08 (s, 0.40*1H, CH-triazole), 8.41 and 8.44 (br. s, 2H); ^{13}C NMR (100 MHz, DMSO-d_6) δ 10.9, 15.1, 17.5, 22.6, 23.2, 23.8, 33.8, 35.7, 41.5, 42.4, 45.7, 50.0, 51.6, 52.9, 55.2, 55.6, 57.4, 62.7, 98.3, 103.0 (d, $^2J_{C\text{-}F}$ = 24.7 Hz), 104.5, 109.4 (d, $^2J_{C\text{-}F}$ = 28.4 Hz), 110.9, 111.1, 117.8, 123.2, 124.8, 128.8, 132.6, 133.2, 141.1, 156.8, 157.2 (d, $^1J_{C\text{-}F}$ = 236.6 Hz), 159.9, 169.1, 171.3, 173.3; ^{19}F (188 MHz, $CDCl_3$) δ -46.51 - -45.26 (m, F, Ar); Anal. Calcd for $C_{40}H_{57}Cl_2FN_8O_6{\cdot}3H_2O$ (889.8805): C, 53.99; H, 7.14; found: C, 54.57; H, 7.20.

L-Isoleucyl-*N*²-(2,4-dimethoxybenzyl)-*N*¹-{(1*S*)-2-[4-({[3-(2,8-dimethyl-1,2,3,4-tetrahydro-5*H*-pyrido[4,3-*b*]indol-5-yl)propanoyl]oxy}methyl)-1*H*-1,2,3-triazol-1-yl]-1-methylethyl}-2-methylalaninamide dihydrochloride (mixture of rotamers, ~ 1.5:1) (7c): According to the general procedure **7c** was obtained from **6c** as a white solid in 96% yield; mp 138 °C; $[\alpha]_D^{25}$ +1.8 (*c* 0.3, MeOH); ^{1}H NMR (DMSO-d_6, 400 MHz) δ 0.73-0.85 (m, 6H, 2 CH_3), 0.96-1.02 (m, 1H, CH), 1.07 (d, J = 6.6 Hz, 3H, CH_3), 1.17 (s, 3H, CH_3), 1.21 (s, 3H, CH_3), 1.42-1.53 (m, 1H, CH_2), 1.82-1.91 (m, 1H, CH_2), 2.35 and 2.36 (s, 3H, CH_3), 2.64-2.79 (m, 2H, CH_2), 2.90 and 2.91 (s, 3H, CH_3), 3.07-3.24 (m, 1H, CH_2), 3.36-3.47 (m, 1H, CH_2), 3.53-3.57 (m, 1H, CH_2), 3.64-3.69 (m, 1H, CH_2), 3.74, 3.75, 3.78 and 3.81 (s, 6H, 2 OCH_3), 4.13-4.39 (m, 9H), 4.46-4.58 (m, 2H), 4.75 (d, J_{AB} = 18.8 Hz, 1H, CH_2),5.03-5.07 (m, 1H, NH), 6.48-6.52 (m,

1H, Ar), 6.56-6.60 (m, 1H, Ar), 6.92-7.00 (m, 1H, Ar), 7.17-7.21 (m, 1H, Ar), 7.42-7.53 (m, 2H, Ar), 7.92 (s, 0.60*1H, CH-triazole), 8.06 (s, 0.40*1H, CH-triazole), 8.41 and 8.44 (br. s, 2H); ^{13}C NMR (100 MHz, DMSO-d_6) δ 11.0, 15.1, 17.5, 21.1, 22.6, 23.2, 23.8, 33.9, 35.7, 41.5, 42.4, 45.7, 49.5, 50.1, 51.6, 52.9, 55.2, 55.6, 57.4, 62.7, 98.3, 101.2, 104.5, 109.6, 117.3, 117.8, 123.0, 124.8, 125.3, 128.1, 128.8, 131.0, 134.3, 141.1, 156.8, 159.9, 169.1, 171.3, 173.3; Anal. Calcd for $C_{41}H_{60}Cl_2N_8O_6 \cdot 3H_2O$ (885.9167): C, 55.59; H, 7.51; found: C, 55.95; H, 6.91.

L-Isoleucyl-N^2-(2,4-dimethoxybenzyl)-N^1-{(1*S*)-2-[4-({[3-(8-methoxy-2-methyl-1,2,3,4-tetrahydro-5*H*-pyrido[4,3-*b*]indol-5-yl)propanoyl]oxy}methyl)-1*H*-1,2,3-triazol-1-yl]-1-methylethyl}-2-methylalaninamide dihydrochloride (mixture of rotamers) (7d): According to the general procedure **7d** was obtained from **6d** as a white solid in 94% yield; mp 159 °C; $[\alpha]_D^{20}$ +4.2 (*c* 0.5, MeOH); ^{1}H NMR (DMSO-d_6, 400 MHz) δ 0.71-0.84 (m, 6H, 2 CH_3), 0.94-1.02 (m, 1H, CH), 1.09 (d, J = 6.0 Hz, 3H, CH_3), 1.15 (s, 3H, CH_3), 1.17 (s, 3H, CH_3), 1.43-1.54 (m, 1H, CH_2), 1.78-1.89 (m, 1H, CH_2), 2.67-2.78 (m, 2H, CH_2), 2.90 and 2.91 (s, 3H, CH_3), 3.10-3.21 (m, 2H, CH_2), 3.37-3.55 (m, 2H, CH_2), 3.63-3.71 (m, 2H, CH_2), 3.74 (s, 6H, 2 OCH_3), 3.78 and 3.81 (s, 3H, OCH_3), 4.08-4.23 (m, 2H), 4.26-4.40 (m, 4H), 4.46-4.61 (m, 3H), 4.75 (d, J_{AB} = 18.4 Hz, 1H, CH_2), 5.05 (br. s, 1H, NH), 6.50 (d, J = 8.9 Hz, 1H, Ar), 6.57 (br. s, 1H, Ar), 6.73-6.79 (m, 1H, Ar), 6.93-7.00 (m, 1H, Ar), 7.44-7.52 (m, 2H, Ar), 8.07 and 8.09 (s, 1H, CH-triazole), 8.46 (br. s, 2H); ^{13}C NMR (100 MHz, DMSO-d_6) δ 10.9, 15.1, 17.5, 22.6, 22.3, 23.7, 33.9, 35.7, 38.6, 41.5, 42.4, 45.7, 49.6, 50.1, 52.9, 54.7, 55.2, 55.4, 55.6, 57.4, 62.7, 98.3, 100.1, 101.4, 104.5, 110.5, 111.1, 117.8, 125.0, 125.3, 128.8, 130.9, 131.5, 141.1, 153.7, 156.8, 159.9, 169.2, 170.6, 173.3; Anal. Calcd for $C_{41}H_{60}Cl_2N_8O_7 \cdot 3H_2O$ (901.9161): C, 54.60; H, 7.38; found: C, 55.12; H, 7.50.

L-Seryl-N^2-(2,4-dimethoxybenzyl)-N^1-((1*S*)-1-{[4-({[3-(2,8-dimethyl-1,2,3,4-tetrahydro-5*H*-pyrido[4,3-*b*]indol-5-yl)propanoyl]oxy}methyl)-1*H*-1,2,3-triazol-1-yl]methyl}-2-methylpropyl)glycinamide dihydrochloride (mixture of rotamers, ~ 1.5:1) (7e): According to the general procedure **7e** was obtained from **6e** as a white solid in 95% yield; mp 130 °C; $[\alpha]_D^{25}$ -4.5 (*c* 0.1, MeOH); ^{1}H NMR (DMSO-d_6, 400 MHz) δ 0.85-0.95 (m, 6H, 2 CH_3), 1.65-1.77 (m, 1H, CH), 2.36 (s, 3H, CH_3), 2.68-2.79 (m, 2H, CH_2), 2.90 and 2.91 (s, 3H, CH_3), 3.07-3.22 (m, 2H, CH_2), 3.37-3.59 (m, 4H, 2 CH_2), 3.65-3.80 (m, 2H, CH_2, 6H, 2 OCH_3), 3.85-3.93 (m, 1H), 3.96-4.08 (m, 2H), 4.14-4.23 (m, 2H), 4.27-4.36 (m, 3H), 4.45-4.57 (m, 4H), 5.03-5.12 (m, 1H, NH), 6.41-6.59 (m, 2H, Ar), 6.93-7.05 (m, 2H, Ar), 7.17-7.21 (m, 1H, Ar),

7.30-7.38 (m, 1H, Ar), 7.96 (s, 0.60*1H, CH-triazole), 8.04 (s, 0.40*1H, CH-triazole), 8.27 and 8.41 (br. s, 2H); ^{13}C NMR (100 MHz, DMSO-d_6) δ 17.8, 18.0, 19.0, 19.3, 21.1, 29.8, 33.9, 38.5, 44.0, 46.6, 49.5, 50.1, 51.6, 52.6, 54.4, 54.9, 55.3, 55.6, 60.2, 98.2, 98.6, 101.2, 104.5, 109.6, 115.9, 117.3, 123.0, 124.8, 125.5, 128.1, 131.0, 132.8, 134.3, 141.1, 158.4, 160.0, 167.1, 170.6, 171.3; Anal. Calcd for $C_{38}H_{54}Cl_2N_8O_7 \cdot 2H_2O$ (841.821): C, 54.22; H, 6.94; found: C, 53.52; H, 6.29.

L-Phenylalanyl-*N*^{2}-(2,4-dimethoxybenzyl)-*N*^{1}-{(1*S*)-2-[4-({[3-(2,8-dimethyl-1,2,3,4-tetrahydro-5*H*-pyrido[4,3-*b*]indol-5-yl)propanoyl]oxy}methyl)-1*H*-1,2,3-triazol-1-yl]-1-methylethyl}-2-methylalaninamide dihydrochloride (mixture of rotamers, ~ 1.5:1) (7f): According to the general procedure **7f** was obtained from **6f** as a white solid in 90% yield; mp 145 °C; $[\alpha]_D^{25}$ +3.0 (*c* 0.1, MeOH); ^{1}H NMR (DMSO-d_6, 400 MHz) δ 1.06-1.18 (m, 9H, 3 CH_3), 2.34 and 2.36 (s, 3H, CH_3), 2.64-2.79 (m, 2H, CH_2), 2.82-2.88 (m, 1H, CH_2), 2.90 and 2.91 (s, 3H, CH_3), 3.07-3.21 (m, 3H, 2 CH_2), 3.51-3.56 (m, 1H, CH_2), 3.63-3.68 (m, 1H, CH_2), 3.71, 3.73, 3.75, 3.79 (s, 6H, 2 OCH_3), 3.96-4.01 (m, 2H), 4.26-4.64 (m, 10H), 5.02-5.07 (m, 1H, NH), 6.22-6.28 (m, 1H, Ar), 6.53-6.59 (m, 1H, Ar), 6.70-6.81 (m, 1H, Ar), 6.91-6.99 (m, 1H, Ar), 7.02-7.09 (m, 2H, Ar), 7.16-7.22 (m, 1H, Ar), 7.26-7.32 (m, 4H, Ar), 7.95 (s, 0.60*1H, CH-triazole), 8.09 (s, 0.40*1H, CH-triazole), 8.61 and 8.67 (br. s, 2H); ^{13}C NMR (100 MHz, DMSO-d_6) δ 17.7, 21.1, 23.1, 23.3, 23.9, 33.9, 36.8, 38.5, 41.5, 42.8, 45.6, 49.6, 50.1, 51.6, 51.8, 53.0, 54.9, 55.2, 55.6, 56.0, 57.4, 62.6, 98.2, 101.2, 104.6, 109.5, 117.3, 117.5, 123.1, 124.8, 125.4, 127.3, 128.1, 128.7, 129.7, 131.1, 134.3, 135.0, 141.0, 156.8, 159.7, 168.7, 171.3, 173.1; Anal. Calcd for $C_{44}H_{58}Cl_2N_8O_6 \cdot H_2O$ (883.9023): C, 59.79; H, 6.84; found: C, 60.30; H, 7.14.

L-Alanyl-*N*^{1}-{2-[4-({[3-(2,8-dimethyl-1,2,3,4-tetrahydro-5*H*-pyrido[4,3-*b*]indol-5-yl)propanoyl]oxy}methyl)-1*H*-1,2,3-triazol-1-yl]ethyl}-*N*^{2}-(4-methoxybenzyl)-2-methylalaninamide dihydrochloride (mixture of rotamers, ~ 1.5:1) (7g): According to the general procedure **7g** was obtained from **6g** as a white solid in 95% yield; mp 133 °C; $[\alpha]_D^{25}$ +2.5 (*c* 0.2, MeOH); ^{1}H NMR (DMSO-d_6, 400 MHz) δ 1.14-1.30 (m, 9H, 3 CH_3), 2.34 and 2.35 (s, 3H, CH_3), 2.65-2.80 (m, 2H, CH_2), 2.90 and 2.91 (s, 3H, CH_3), 3.07-3.25 (m, 2H, CH_2), 3.37-3.47 (m, 4H, 2 CH_2), 3.53-3.56 (m, 2H, CH_2), 3.65-3.70 (m, 1H), 3.73 and 3.74 (s, 3H, OCH_3), 4.04-4.23 (m, 3H), 4.25-4.35 (m, 3H), 4.64 (br. s, 2H), 5.07-5.10 (m, 1H, NH), 6.89-7.00 (m, 3H, Ar), 7.18-7.21 (m, 1H, Ar), 7.32-7.39 (m, 3H, Ar), 7.98 (s, 0.60*1H, CH-triazole), 8.11 (s, 0.40*1H, CH-triazole), 8.44 and 8.46 (br. s, 2H); ^{13}C NMR (100 MHz, DMSO-d_6) δ 16.7, 21.1,

23.6, 24.0, 33.9, 38.5, 41.5, 44.3, 46.9, 47.1, 48.4, 49.5, 50.1, 51.6, 54.9, 55.1, 62.6, 101.2, 109.6, 113.9, 117.3, 123.1, 123.3, 124.8, 125.3, 128.0, 129.9, 131.1, 134.4, 141.2, 158.4, 170.1, 171.3, 173.9; Anal. Calcd for $C_{36}H_{50}Cl_2N_8O_5 \cdot 4H_2O$ (817.7996): C, 52.87; H, 7.15; found: C, 52.15; H, 7.11.
